# Femoro-Femoral Bypass for a Vascular Complication During Transfemoral Aortic Valve Implantation

**DOI:** 10.7759/cureus.81239

**Published:** 2025-03-26

**Authors:** Kazunori Omote, Takeshi Kamada, Makoto Furugen, Shunsuke Ohori, Azusa Furugen, Daisuke Sunaga, Naohiro Funayama

**Affiliations:** 1 Department of Cardiology, Hokkaido Cardiovascular Hospital, Sapporo, JPN; 2 Department of Cardiothoracic Surgery, Hokkaido Cardiovascular Hospital, Sapporo, JPN

**Keywords:** aortic stenosis, endovascular therapy, femoro-femoral bypass, transcatheter aortic valve implantation, vascular complication

## Abstract

A 94-year-old woman underwent transcatheter aortic valve implantation (TAVI) for treatment of severe aortic stenosis (AS). Final angiography showed the right external iliac artery occlusion due to vascular injury related to a large-diameter introducer sheath. Although we performed endovascular therapy, no guidewires could cross the culprit lesion because the intima had frapped and occluded the vessel lumen. Therefore, we performed femoro-femoral bypass for bailout of acute limb ischemia. Femoro-femoral bypass is a less invasive, shorter operation time and therefore a reasonable strategy as an urgent bailout for vascular complications during TAVI in super-aged or higher-risk surgical patients with severe AS.

## Introduction

The number of patients with heart failure (HF) is increasing due to the aging of the population, along with therapeutic innovations in the management of cardiovascular diseases [1]. Approximately one-third of patients with HF have some valvular heart disease [2]. Among them, aortic stenosis (AS) is the most common valvular heart disease, especially in the elderly. As its treatments, transcatheter aortic valve implantation (TAVI) is recognized as an established, less-invasive strategy for higher surgical risk patients with symptomatic severe AS.

While advances in technology have made it possible to lower the profile of the valve delivery system from the femoral artery, sheath-related vascular complications still increase morbidity and mortality in patients who undergo transfemoral TAVI [3, 4]. Thus, it is important for the heart team to evaluate the access site with reference to pre-procedural enhanced computed tomography (CT) findings and to manage vascular complications during the TAVI procedure.

This is a successful bailout case for acute limb ischemia due to a vascular complication during transfemoral TAVI by femoro-femoral bypass surgery in an elderly patient with severe AS.

## Case presentation

**Figure 1:**
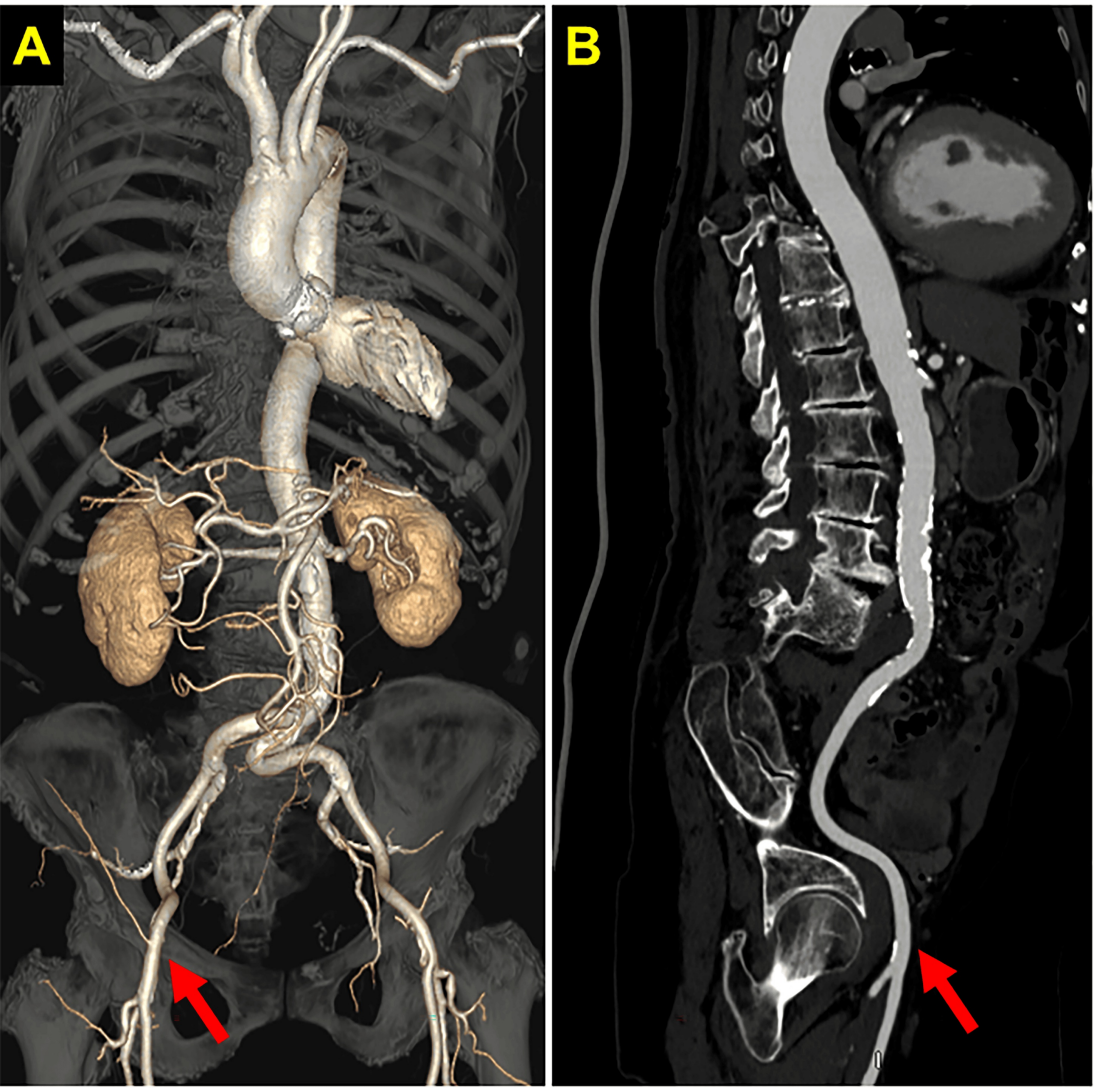
Findings of pre-procedural enhanced computed tomography Red arrows indicate the access site.

A 94-year-old woman with a medical history of hypertension and dyslipidemia was referred to our hospital for treatment of acute decompensated heart failure (ADHF) due to severe AS. After the treatment of ADHF, the heart team decided to perform TAVI for this patient. On the pre-procedural contrast CT, significant stenosis was not observed in any access sites (Figures 1A, 1B).

**Figure 2:**
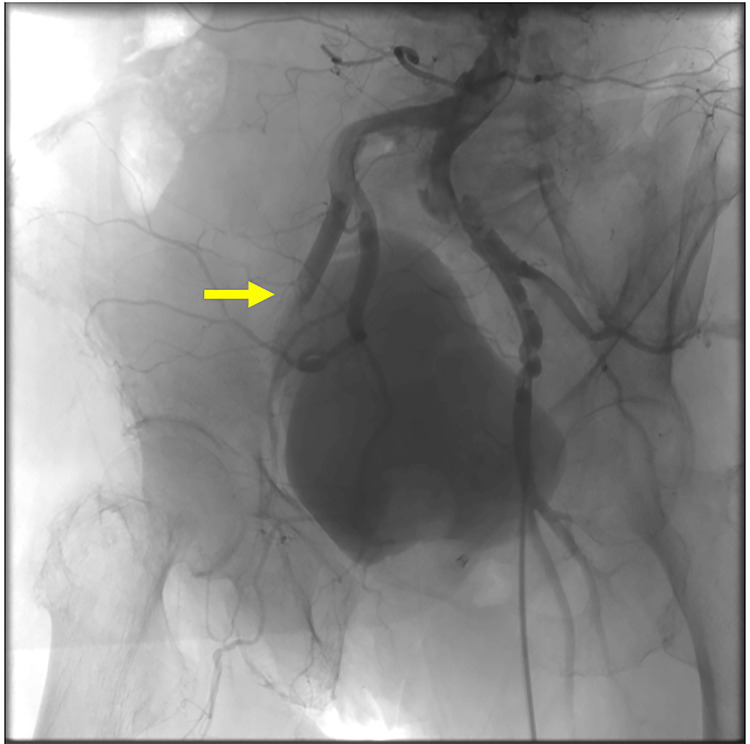
Final angiography at the access site Yellow arrow indicates the occlusion site at right external-iliac artery.

Thus, transfemoral-TAVI was performed via the right side, and a dry seal introducer sheath (W.L. Gore & Associates, Inc., Flagstaff, AZ, USA) was inserted. A 26-mm Evolut FX valve (Medtronic, Minneapolis, MN, USA) was delivered and deployed successfully. However, final angiography at the access site showed the right external iliac artery occlusion (Figure 2).

**Figure 3:**
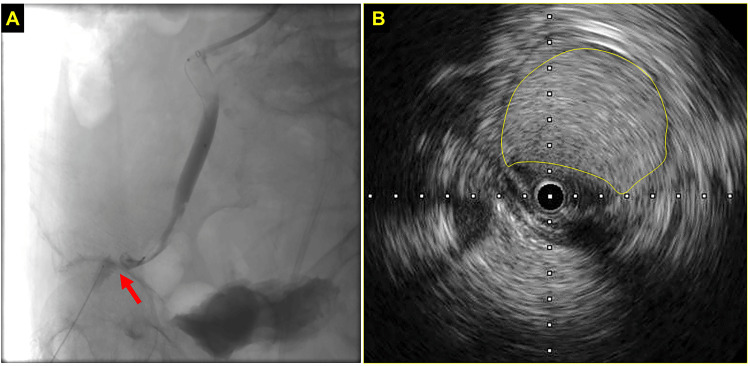
Endovascular therapy during TAVI (A) and the finding of intravascular ultrasound (B) The red arrow indicates the occluded site of the vessel lumen. The yellow circle indicates the frapped intima revealed by intravascular ultrasound. TAVI: transcatheter aortic valve implantation

Then, we performed endovascular therapy (EVT) by a bi-directional approach from an antegrade 6-Fr guiding system via the left brachial artery and a retrograde microcatheter via the right femoral artery. However, the guidewires from antegrade or retrograde could not cross the culprit lesion. Intravascular ultrasound (IVUS) showed that the intima had frapped and completely occluded the vessel lumen (Figures 3A, 3B).

**Figure 4:**
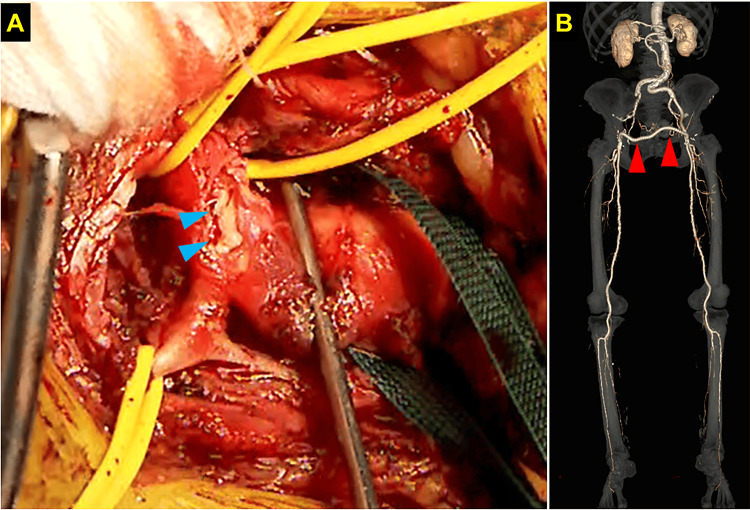
Macroscopic findings (A) and post-procedural enhanced computed tomography (B) Blue arrows indicate the ruptured intima in the culprit lesion. Red arrows indicate the femoro-femoral bypass graft.

Therefore, we had to change our plan to a surgical procedure. Since aorto-femoral bypass carries a higher risk of surgery for this super-aged patient, we selected femoro-femoral bypass for the bailout of acute limb ischemia. When the vessel was opened, a ruptured intima was observed in the culprit lesion (Figure 4A). The graft GORE PROPATEN (W. L. Gore & Associates, Inc.) 6 mm was tunneled from the left groin incision to the right and anastomosed to each vessel (Figure 4B).

We successfully treated acute lower extremity ischemia via femoro-femoral bypass. This patient was discharged home after surgery without lower limb amputation.

## Discussion

Many elderly patients with symptomatic severe AS cannot undergo surgical aortic valve replacement (SAVR) because of the high surgical risk [5]. Transcatheter aortic valve implantation is a less invasive alternative to SAVR and is recognized as a well-established therapeutic strategy for treating symptomatic severe AS, regardless of intermediate or even high operative risk [6]. However, there are specific complications related to TAVI. Among them, sheath-related vascular complications are associated with worse clinical outcomes in patients who underwent TAVI [3,4].

In case of vascular complications during the TAVI procedure, patients are usually treated by EVT with balloon angioplasty, stents, or covered stents, but some cases require surgical vessel repair [7]. In this case, we first selected to perform EVT. But any guidewires could not cross the culprit lesion because the flapped intima completely covered the vessel lumen. And thus, we decided to perform surgical repair as a secondary option.

Because long-term graft patency is superior after aorto-femoral bypass as compared with femoro-femoral bypass, open aorto-femoral bypass surgery is still the gold standard for aortoiliac reconstruction [8]. According to a previous comparative study, three-year graft patency was inferior after femoro-femoral bypass as compared with aorto-femoral bypass (60% versus 85%), although both surgeries achieved limb salvage in more than 85% of patients at three years [8]. But femoro-femoral bypass is a less invasive, shorter operation time (within one hour in this case), and therefore a reasonable strategy as an ‘urgent bailout’ for vascular complications during the TAVI procedure in super-aged or higher operative risk patients with severe AS.

## Conclusions

We experienced a successful bailout case of acute limb ischemia due to sheath-related vascular injury during transfemoral TAVI via femoro-femoral bypass. Endovascular therapy is the first choice for a bailout of this complication. But femoro-femoral bypass is another acceptable option as an urgent bailout for vascular complications during the TAVI procedure in super-aged or high surgical risk patients.
